# Tissue-resident memory T cells in urinary tract diseases

**DOI:** 10.3389/fimmu.2025.1535930

**Published:** 2025-02-24

**Authors:** Guofeng Xu, Yuying Li, Guanting Lu, Daoyuan Xie

**Affiliations:** ^1^ Inflammation and Allergic Diseases Research Unit, The Affiliated Hospital of Southwest Medical University, Luzhou, China; ^2^ Department of Respiratory Critical Care, The Affiliated Hospital of Southwest Medical University, Luzhou, China; ^3^ Laboratory of Translational Medicine Research, Deyang People’s Hospital of Chengdu University of Traditional Chinese Medicine, Deyang, China

**Keywords:** tissue-resident memory T cells, urinary tract, infection, tumor, immunotherapy

## Abstract

Tissue-resident memory T (T_RM_) cells are a specialized subset of memory T cells that permanently reside in non-lymphoid tissues, providing localized and long-lasting immune protection. In the urinary tract, T_RM_ cells play critical roles in defending against infections, mediating tumor immunity, and influencing the pathogenesis of chronic inflammatory diseases. Their therapeutic potential is immense, with promising avenues for vaccine development, enhanced cancer immunotherapy, and targeted treatments for chronic inflammation. However, challenges remain in harnessing their protective roles while minimizing their pathological effects, particularly in immunosuppressive or inflammatory microenvironments. This review explores the diverse roles of T_RM_ cells in urinary tract diseases, including infections, cancer, and chronic inflammation, and discusses therapeutic strategies and future directions for leveraging T_RM_ cells to improve clinical outcomes. By advancing our understanding of T_RM_ cell biology, we can develop innovative interventions that balance their immune-protective and regulatory functions.

## 1 Introduction

Tissue-resident memory T (T_RM_) cells represent a unique subset of memory T cells that reside permanently within non-lymphoid tissues without recirculating through the blood or lymphatic systems (1). Unlike central or effector memory T cells, T_RM_ cells are characterized by the expression of markers such as CD69, which prevents egress from tissues, and CD103, which promotes adhesion to epithelial cells (2–4). These cells serve as sentinels of localized immunity, poised to rapidly respond to reinfection by previously encountered pathogens (5, 6). This unique population comprises two main subsets: CD4^+^ and CD8^+^ T_RM_ cells, each with distinct characteristics, developmental pathways, and functional roles (7). CD8^+^ T_RM_ cells act as the first line of defense against viral re-infections, rapidly eliminating infected cells upon pathogen re-encounter (8). In contrast, CD4^+^ T_RM_ cells play a critical role in coordinating broader immune responses, including the support of B cell activity and the maintenance of local immunity at various tissue sites, such as skin and mucosal surfaces (9). Recent studies have shown that T_RM_ cells comprise approximately 10-30% of the total T cell population in non-lymphoid tissues, with variations depending on tissue type and disease state (10). For example, in inflamed tissues, T_RM_ cells can account for up to 60% of the T cell population, while in cancer tissues, their proportion typically ranges from 5% to 20%, depending on the tumor microenvironment (11–13). This variability in T_RM_ cells frequency underscores their dynamic roles in immune responses across different conditions. Furthermore, T_RM_ cells have been shown to persist in non-lymphoid tissues for extended periods, with some studies reporting survival times ranging from several months to years. This long-term presence highlights their critical role in maintaining tissue immunity (14, 15). These distinct functions underscore the importance of understanding the heterogeneity of T_RM_ cells in the context of urinary tract diseases, setting the stage for a more detailed exploration of their roles in infections, tumors, and chronic inflammation that follows.

The urinary tract is a critical site for immune responses, balancing pathogen defense with tolerance of non-harmful antigens. It comprises the urinary and reproductive systems, including the kidneys, bladder, and genital organs. T_RM_ cells, identified in bladder, ovarian, vaginal, kidney, uterine, prostate, and penile tissues, are vital for local immunity against infections and tumors and may modulate autoimmune responses (16–21). Beyond the urinary tract, T_RM_ cells have also been implicated in other cancer types, such as melanoma, lung cancer, and breast cancer, where they contribute to tumor surveillance and immune control (22–24). For instance, in melanoma, high densities of CD8^+^ T_RM_ cells are associated with improved patient survival and response to immunotherapy (25). Similarly, in lung cancer, T_RM_ cells have been shown to enhance anti-tumor immunity and predict favorable clinical outcomes (26). These findings highlight the broader relevance of T_RM_ cells in cancer immunity and underscore the need for further research into their roles across different tissue types and disease contexts. Their strategic localization within the epithelial barriers and ability to interact with other immune and stromal cells underline their importance as both defenders and regulators of tissue health. Understanding the unique biology of T_RM_ cells in this context is vital for developing targeted therapeutic strategies.

## 2 The role of T_RM_ cells in urinary tract infection

T_RM_ cells in the urinary tract share features with those in other parts of the body, such as the expression of CD69 and CD103, as well as the characteristic of tissue residency. They also display unique adaptations to their local microenvironments, including their positioning in specific tissues-like the lamina propria and basal epithelial lining in vaginal and penile tissues-and the expression of molecular markers such as CXCR3, P2RX7, CXCR6, and CD49a (27–31). Immediately upon entry, these adaptations indicate their strategic positioning in barrier tissues to intercept pathogens, including bacteria, viruses, and fungi.

Urinary tract infections (UTIs) are among the most common infections globally, characterized by frequent recurrence despite antibiotic treatment (32, 33). T_RM_ cells play a pivotal role in the immune defense against these infections by establishing localized, long-term immunity in the urinary tract. In response to primary infection, pathogen-specific T_RM_ cells are generated and retained in the bladder mucosa, where they can rapidly detect and respond to reinfection. However, T_RM_ cells, including CD4^+^ and CD8^+^ subsets, do not directly recognize pathogens but instead rely on interactions with antigen-presenting cells (APCs) to initiate their immune responses. Upon reinfection, dendritic cells (DCs) and macrophages in the urinary tract rapidly process and present pathogen-derived antigens via MHC class II and class I molecules, respectively. CD4^+^ T_RM_ cells recognize antigens presented on MHC class II by APCs, leading to their activation and subsequent secretion of cytokines, such as interferon-gamma (IFN-γ) and tumor necrosis factor-alpha (TNF-α), which recruit and activate additional immune cells like neutrophils and macrophages (34). Similarly, CD8^+^ T_RM_ cells are activated through the recognition of antigens presented on MHC class I by APCs, enabling them to exert cytotoxic effects via the release of granzymes and perforin (35). This interaction between T_RM_ cells and APCs ensures a rapid and localized immune response that is essential for the effective clearance of pathogens during secondary infections. These cells express markers such as CD69, which anchors them within tissues, and secrete cytokines like IFN-γ upon activation, initiating a cascade of immune responses that recruit neutrophils and other effector cells to the site of infection (18). T_RM_ cells are particularly effective in combating uropathogenic *Escherichia coli* (UPEC), the most common causative agent of UTIs. CD4^+^ and CD8^+^ T_RM_ cells play complementary roles in the immune defense against UTIs. CD4^+^ T_RM_ cells primarily facilitate immune responses by secreting cytokines that help activate other immune cells, such as macrophages and B cells, promoting antibody production and maintaining immune homeostasis (36). These cells also contribute to tissue repair and inflammation regulation in the urinary tract. In contrast, CD8^+^ T_RM_ cells exert direct cytotoxic effects by killing infected epithelial cells through the release of granzymes and perforin, limiting pathogen replication (37). Both CD4^+^ and CD8^+^ T_RM_ cells provide long-term immunity by persisting in the bladder mucosa, enabling rapid and effective responses to reinfection. However, the activation and persistence of T_RM_ cells, especially during recurrent UTIs, can also lead to chronic inflammation and tissue damage, emphasizing the need for therapeutic strategies that balance immune protection with the prevention of excessive tissue injury.

In the context of secondary infections, T_RM_ cells are poised to rapidly exert their effector functions. They produce granzyme B and pro-inflammatory cytokines that serve to recruit and activate additional immune cells. This swift response is essential for controlling viral reactivation and combating bacterial infections. Studies have shown that UPEC-specific T_RM_ cells remain in the bladder mucosa long after the initial infection has resolved, providing a robust immunological memory that reduces the severity of subsequent infections (18). However, the persistence and activation of T_RM_ cells can also contribute to tissue damage and inflammation in the bladder, especially in recurrent infections, potentially exacerbating symptoms. Moreover, their interactions with other immune cells, including macrophages and DCs, further shape the local immune microenvironment (38, 39).

When T_RM_ cells function is impaired-due to factors such as immunosuppressive environments, chronic inflammation, or aging-the immune response becomes less effective (40–42). In such scenarios, pathogen clearance is delayed, leading to prolonged infection, increased pathogen load, and heightened risk of tissue damage. The absence of immediate effector responses also results in a heavier reliance on systemic adaptive immunity, which takes more time to activate and may not effectively localize to the infection site.

## 3 T_RM_ cells in urinary tract tumors

**Figure 1:**
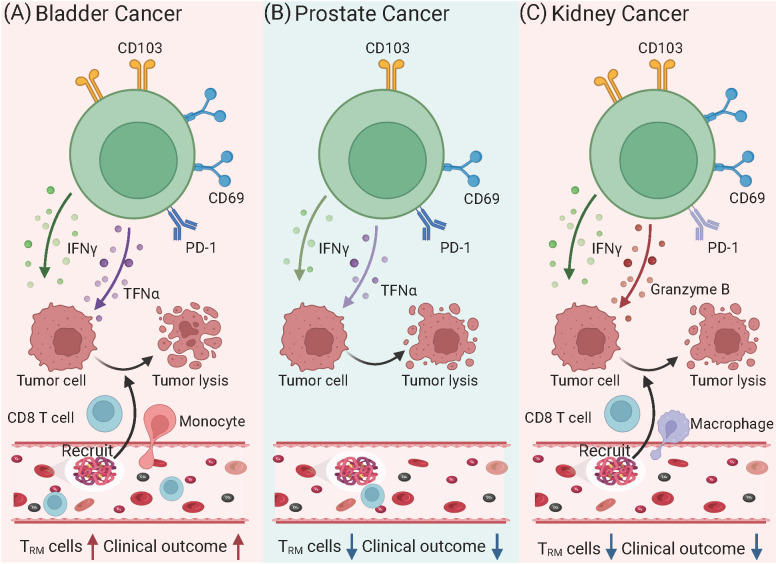
Phenotypic and functional variations of T_RM_ cells in urinary tract tumors. **(A)** Bladder Cancer: T_RM_ cells exhibit high expression of CD103 and CD69, alongside PD-1, indicating a tumor-resident yet partially exhausted state. These cells produce cytokines such as IFN-γ and TNF-α, which enhance anti-tumor immunity by recruiting CD8^+^ T cells and NK cells. Higher densities of CD103^+^CD8^+^ T_RM_ cells correlate with improved prognosis and better responses to immune checkpoint inhibitors targeting PD-1/PD-L1. However, persistent antigen exposure may lead to progressive exhaustion, reducing their cytotoxic potential. **(B)** Prostate Cancer: T_RM_ cells show reduced CD103 and limited effector functions due to an immunosuppressive tumor microenvironment. Elevated levels of TGF-β and myeloid-derived suppressor cells contribute to T_RM_ cells dysfunction, reducing their ability to mount effective anti-tumor responses. As a result, T_RM_ cells in prostate cancer are often associated with poor immune control and tumor progression. Moreover, their diminished cytotoxic potential limits the effectiveness of PD-1 blockade therapy in prostate cancer compared to other tumors. **(C)** Kidney Cancer: T_RM_ cells in renal cell carcinoma express high levels of CD103 and CD69, often accompanied by PD-1, suggesting a dual role in tumor surveillance and immune suppression. Despite retaining the capacity to produce IFN-γ, TNF-α, and Granzyme B, T_RM_ cells frequently become functionally exhausted in the TME, reducing their ability to control tumor growth. However, high T_RM_ cells infiltration is associated with better prognosis and enhanced responses to ICIs, particularly PD-1 inhibitors. Depletion studies in murine models suggest that T_RM_ cells play a crucial role in mediating the efficacy of immunotherapies in RCC.

T_RM_ cells are emerging as critical players in the immune response to urinary tract tumors, including bladder, prostate, and kidney cancers (**Figure 1**). These cells reside within tumor tissues, where they exert localized anti-tumor effects by recognizing and responding to tumor-specific antigens. T_RM_ cells contribute to immune surveillance, influence the tumor microenvironment (TME), and enhance responses to immune checkpoint inhibitors (ICIs). In urinary tract tumors, T cell exhaustion is a well-known feature of the tumor immune microenvironment, characterized by the upregulation of inhibitory receptors such as PD-1, TIM-3, and LAG-3 (43). Interestingly, while T_RM_ cells are generally associated with robust immune responses, they are not immune to the effects of exhaustion in tumor settings. T_RM_ cells within the TME can become exhausted over time due to chronic antigen stimulation and the immunosuppressive signals present in the tumor. This exhaustion is associated with a reduced capacity for cytokine production, cytotoxic activity, and overall immune surveillance (44). However, unlike recirculating T cells, T_RM_ cells may maintain some degree of function due to their unique tissue-residency markers, such as CD69 and CD103. These markers help anchor T_RM_ cells in the tissue, potentially allowing them to persist in the TME even when other T cells become exhausted. Recent studies suggest that enhancing the functionality of exhausted T_RM_ cells in tumors could improve responses to immunotherapies, such as ICIs, by restoring their effector functions (45).

In urinary cancers, tumor-infiltrating T cells display a heterogeneous phenotype. The majority of these cells express CD69 in prostate and kidney cancers, while in bladder cancers, they also commonly express CD103. Understanding the role of T_RM_ cells in these cancers offers valuable insights into novel immunotherapeutic strategies and their potential to improve clinical outcomes.

### 3.1 T_RM_ cells in bladder cancer

In bladder cancer, T_RM_ cells play a pivotal role in shaping anti-tumor immunity within the TME (46). T_RM_ cells are localized in the bladder epithelium and tumor tissues, where they exhibit hallmark features such as CD69 and CD103 expression, enabling them to remain anchored in the tissue and maintain prolonged immune surveillance (47). These cells respond to tumor-specific antigens by producing effector cytokines like IFN-γ and tumor necrosis factor-alpha (TNF-α), which stimulate cytotoxic activity and enhance the recruitment of other immune cells, such as CD8^+^ T cells and natural killer cells, to the tumor site.

Recently, an in-depth analysis of CD103^+^CD8^+^ T_RM_ cells within muscle-invasive bladder cancer (MIBC) tissues has unveiled a significant correlation with improved overall survival outcomes. The study demonstrated that patients with a high infiltration of CD103^+^CD8^+^ T_RM_ cells, rather than CD8^+^ T cells alone, are more likely to benefit from both immunotherapy and adjuvant chemotherapy (ACT). These T_RM_ cells are associated with an enhanced IFNγ-enriched and T cell-inflamed antitumor microenvironment (47). The findings underscore the pivotal role of CD103^+^CD8^+^ T_RM_ cells in antitumor immunity and their potential as an optimal prognostic biomarker, serving as a superior companion predictor for treatment responses to PD-L1 inhibitors and ACT in MIBC patients. T_RM_ cells may also act as a biomarker for predicting immunotherapy efficacy (48). CD103^+^CD8^+^ T_RM_ cells despite frequently exhibiting elevated levels of immune checkpoint molecules such as PD-1, TIM-3, and LAG-3, retain their capacity to produce cytotoxic molecules and effector cytokines, which is notably different from their CD103-negative counterparts (49, 50). This characteristic is particularly relevant in the context of renal cell carcinoma, bladder, and ovarian cancer, where the high expression of PD-1 on CD103^+^ T_RM_ cells is thought to be a key factor in the efficacy of anti-PD-1 therapies (51–53). Evidence from murine tumor models indicates that the depletion of CD103^+^ cells results in reduced effectiveness of checkpoint inhibitor treatments, highlighting the importance of these T_RM_ cells in the response to cancer immunotherapy (54).

### 3.2 T_RM_ cells in prostate cancer

In prostate cancer, the role of T_RM_ cells is less well-characterized compared to other urinary tract tumors, but emerging evidence suggests they contribute to both tumor suppression and immune modulation within the prostate microenvironment (20). However, prostate cancer is often associated with a highly immunosuppressive microenvironment that limits T_RM_ cells functionality (55). Elevated levels of regulatory T cells, myeloid-derived suppressor cells, and inhibitory cytokines, such as transforming growth factor-beta (TGF-β), suppress T_RM_ cells activation and reduce their anti-tumor efficacy (56). Clinical studies have confirmed that autologous active cellular immunotherapy resulted in a modest improvement in survival in prostate cancer patients, extending it by approximately 4 months (57). Additionally, androgen deprivation therapy (ADT), a common treatment for prostate cancer, has been shown to alter the immune landscape of the prostate, potentially affecting the generation and activity of T_RM_ cells (58).

### 3.3 T_RM_ cells in kidney cancers

In kidney cancers, particularly renal cell carcinoma (RCC), T_RM_ cells play a critical role in tumor immune surveillance. RCC is recognized as an immunogenic tumor, marked by significant immune cell infiltration into tumor tissues compared to adjacent normal tissues (59). Among these immune cells, CD8^+^ T_RM_ cells, characterized by the expression of CD69 and CD103, are commonly found and are essential for maintaining long-term immune surveillance within the TME have shown that a high density of CD103^+^ T_RM_ cells is associated with better prognostic outcomes in RCC (60). These cells retain their cytotoxic potential and can quickly respond to tumor antigens by producing effector cytokines such as IFN-γ and granzyme B. Depletion of CD103^+^ T_RM_ cells in murine RCC models leads to accelerated tumor growth, highlighting their vital role in controlling tumor progression.

However, the functionality of T_RM_ cells in RCC is often compromised by the expression of immune checkpoint molecules such as PD-1 (61). These inhibitory signals can suppress the effector functions of T_RM_ cells. ICIs, such as anti-PD-1 therapies, can reverse this suppression, rejuvenating T_RM_ cells activity and enhancing anti-tumor immunity. Murine studies have shown that depletion of CD103^+^ cells abrogates the effectiveness of ICIs, emphasizing that T_RM_ cells are crucial mediators of therapeutic responses to checkpoint blockade in RCC.

Emerging therapies, including cancer vaccines, aim to boost the presence and activity of T_RM_ cells within kidney tumors. By inducing tumor-specific T_RM_ cells, these vaccines could enhance immune responses and work synergistically with ICIs to improve treatment efficacy (62). Ongoing clinical trials investigating the combination of ICIs and T_RM_-enhancing strategies offer promising avenues for improving RCC patient outcomes.

## 4 T_RM_ cells in chronic inflammatory diseases of the urinary tract

The roles of T_RM_ cells in various urinary tract diseases differ significantly. As summarized in **Table 1**, their functions range from immune surveillance in bladder cancer to fibrosis promotion in chronic pyelonephritis. The heterogeneity of T_RM_ cells populations, evidenced by their varied phenotypic and functional profiles within the urinary tract, offers a range of benefits. This diversity is crucial for the immune response to infections and cancers, as well as for the complex dynamics of autoimmune and inflammatory diseases affecting the urinary system. T_RM_ cells infiltration in the kidney positively correlates with disease activity, as indicated by increased serum creatinine, proteinuria, hematuria, and histological scores in patients with lupus nephritis (LN) and antineutrophil cytoplasmic antibody (ANCA) associated glomerulonephritis (GN), as well as in murine systemic lupus erythematosus (SLE) models (61, 63–65). In murine GN models, the number of CD4^+^ T cells predominates over CD8^+^ T cells, whereas in humans, nearly equal numbers of CD4^+^ and CD8^+^ T cells are reported in many studies (66–68). Similarly, in the kidneys of patients and mice with LN, the abundance of CD8^+^ T_RM_ cells was significantly increased (69). Under inflammatory conditions, renal T cells exhibit a tissue-resident phenotype, with CD69 widely expressed on both CD4^+^ and CD8^+^ T cells, indicating their significant role in local immune responses. Additionally, CD103 expression is observed on renal T cells, particularly on CD8^+^ T cells in patients with SLE and in SLE-prone mice (63–65). Mice studies show that both commensal and pathogenic bacteria can trigger kidney inflammation. Infection with *S. aureus*, and *C. albicans* leads to kidney CD4^+^ T_RM_ cells adopting an inflammatory TH17 phenotype, exacerbating disease (64). Activation of kidney T_RM_ cells by cytokines like IL-1β, IL-6, and IL-23 through the JAK-STAT pathway amplifies the inflammatory response. Microbiota may activate T cells with kidney-homing potential in the intestine, and fate-mapping suggests T cells migrate from the intestine to the kidney post-nephritis induction. This migration is driven by S1PR1-dependent egress from the intestine and CCL20-dependent entry into the kidney. The role of these newly infiltrated T cells in disease progression is unclear, but their presence suggests a role for microbiota-driven inflammation in promoting autoimmunity in the kidney.

**Table 1:** Roles of T_RM_ cells in urinary tract diseases.

Disease type	T_RM_ phenotype	Core functions	Regulatory mechanisms
Bladder Cancer	CD103^+^CD8^+^, PD-1^+^	Cytotoxicity, immune cell recruitment	PD-1/PD-L1 inhibition, CXCR3-mediated homing
Prostate Cancer	CD69^+^, low CD103 expression	Limited functionality, immunosuppression	TGF-β suppression, MDSC enrichment
Kidney Cancer	CD103^+^CD8^+^, PD-1^+^	Immune surveillance, cytokine secretion	PD-1-mediated exhaustion
Lupus Nephritis	CD69^+^CD8^+^, CD103^+^	TH17 polarization, pro-inflammatory	JAK-STAT activation, gut-kidney migration
Chronic Pyelonephritis	CD4^+^ dominant, inflammatory phenotype	Fibrosis, tissue damage	Persistent antigen stimulation, IL-6/IL-23

In cases of chronic infections, such as chronic pyelonephritis, the formation and role of T_RM_ cells may differ from acute infections. While T_RM_ cells are typically associated with rapid immune responses to reinfection, their role in chronic infections is more complex. In chronic pyelonephritis, the persistent presence of pathogens and ongoing inflammation may affect the dynamics of T_RM_ cells formation. It is possible that T_RM_ cells are generated and persist in the kidneys during chronic infection, but their function may be impaired or dysregulated due to the prolonged inflammatory environment (70). In such conditions, T_RM_ cells may contribute to the chronic inflammation by promoting tissue damage and fibrosis, potentially leading to a maladaptive immune response. Further research is needed to fully understand the mechanisms underlying T_RM_ cells persistence in chronic infections and their dual role in both protective immunity and tissue pathology.

## 5 Therapeutic implications and future directions

The therapeutic potential of T_RM_ cells in urinary tract diseases, including infections, cancers, and chronic inflammatory conditions, is an area of growing interest (**Table 2**). Harnessing the localized immune response of T_RM_ cells offers several promising strategies for improving treatment outcomes. In the context of infections like UTIs, vaccines designed to generate pathogen-specific T_RM_ cells in mucosal tissues could provide long-lasting protection and reduce recurrence (71, 82). Enhancing T_RM_ cells responses through adjuvants or immune modulators could also improve the efficacy of current vaccines and reduce the burden of recurrent infections (83). By deepening our understanding of T_RM_ cells biology in the context of UTIs, novel interventions can be designed to improve long-term immune protection while minimizing immune-mediated pathology.

**Table 2:** T_RM_ cells as therapeutic targets in urinary tract diseases.

Disease	Potential targets	Therapeutic strategy	Challenges & prospects	Reference
Urinary Tract Infections	IL-15, CD103^+^ T_RM_ cells	Vaccines promoting T_RM_ cells development	Balancing protective immunity and avoiding chronic inflammation	(71, 72)
Bladder Cancer	PD-1/PD-L1, CXCR3	PD-1/PD-L1 inhibitors, local T_RM_ cells enhancement	Preventing immune exhaustion in T_RM_ cells; improving delivery to the tumor microenvironment	(30, 73)
Interstitial Cystitis	Pro-inflammatory cytokines	T_RM_ cells inhibitors	Identifying specific T_RM_ cells populations driving pathology	(74)
Kidney Transplant Rejection	Alloreactive T_RM_ cells, IL-2	T_RM_ cells-specific immunosuppressants	Minimizing systemic side effects; avoiding suppression of protective T_RM_ cells	(75–77)
Renal Cell Carcinoma	PD-1, CD103, IL-15	Checkpoint inhibitors, T_RM_ cells-promoting vaccines	Restoring T_RM_ cells functionality in immunosuppressive tumor environments	(61, 78, 79)
Pelvic Inflammatory Disease	CXCR6, inflammatory T_RM_ cells	Local T_RM_ cells modulation through anti-inflammatory agents	Ensuring effective targeting while preserving necessary immune responses	(80, 81)

In urinary tract cancers, T_RM_ cells have demonstrated a significant role in immune surveillance and anti-tumor immunity. Strategies that aim to boost the function of T_RM_ cells, such as ICIs targeting PD-1/PD-L1 or enhancing their local activation, hold promise for improving response rates to immunotherapy (84, 85). In addition to PD-1/PD-L1 inhibitors, recent studies have also highlighted the importance of other immune checkpoints, such as CXCR3 and IL-15, in regulating the immune response to urinary tract tumors (86). CXCR3, a chemokine receptor, plays a crucial role in the trafficking and retention of effector T cells, including T_RM_ cells, to tumor sites. Its expression on tumor-infiltrating lymphocytes is associated with improved immune surveillance and better responses to immunotherapy (87). Targeting CXCR3 may enhance T cell infiltration into tumors, especially in cancers such as bladder and prostate cancer, where T_RM_ cells are critical for anti-tumor immunity. IL-15 is another key molecule involved in the activation and maintenance of T_RM_ cells. It promotes the survival and function of memory T cells, including both CD4^+^ and CD8^+^ subsets. IL-15-based therapies are being explored as a way to boost T_RM_ cells responses in tumors by enhancing their persistence and effector function. Recent preclinical and clinical studies suggest that IL-15 can be used to improve the efficacy of immunotherapies, particularly in cancers where T_RM_ cells play a central role in local immunity (88). Together with PD-1 blockade, targeting these additional immune checkpoints could lead to more effective immune responses by not only reinvigorating exhausted T cells but also enhancing the recruitment and function of T_RM_ cells within tumors. Additionally, combining these approaches with conventional therapies like chemotherapy or radiation could optimize anti-tumor responses (89).

For chronic inflammatory diseases, targeting T_RM_ cells presents a unique challenge, as their persistent activation can contribute to tissue damage and pain. Therapies that modulate T_RM_ cell activity, such as cytokine blockers or interventions that restore immune homeostasis (e.g. Sparsentan, the dual angiotensin II receptor and endothelin type A receptor antagonist), could reduce T_RM_ cell responses, alleviate symptoms and reduce inflammation without compromising immune protection (69).

Looking forward, research into the specific signaling pathways that regulate T_RM_ cell differentiation, retention, and activation will be crucial for developing targeted therapeutic approaches. While T_RM_ cells are well recognized for their role in defending against acute reinfections, their role in chronic infections, such as tuberculosis, HIV, and chronic pyelonephritis, remains less explored. Future studies should focus on understanding how T_RM_ cells are generated and maintained in chronic infection settings, and how their function may be altered over time in the face of persistent antigen exposure and inflammation. Targeting the modulation of T_RM_ cells in chronic infections could lead to novel therapeutic strategies to enhance long-term immunity without causing excessive tissue damage. Immunotherapies targeting T_RM_ cells in tumors: T_RM_ cells have emerged as critical players in anti-tumor immunity, yet their full potential in cancer immunotherapy is still not fully realized. Future research should explore how to enhance the recruitment, persistence, and functionality of T_RM_ cells in tumors, particularly through combination therapies that target immune checkpoints like PD-1, CXCR3, and IL-15. Investigating how T_RM_ cells interact with the TME and their potential exhaustion during long-term antigen exposure could provide valuable insights into how to prevent or reverse T_RM_ cells exhaustion in cancers. T_RM_ in vaccine development: There is growing interest in developing vaccines that aim to generate long-lasting T_RM_ cells populations at mucosal surfaces, such as the urinary tracts. These vaccines could be crucial for preventing infections like UTIs. Future research should focus on identifying the best strategies for inducing robust T_RM_ cells responses through mucosal vaccination, as well as understanding how T_RM_ cells contribute to vaccine-mediated immunity in both infectious and cancer settings.

## 6 Conclusion

In conclusion, T_RM_ cells play a crucial role in the immune defense of the urinary tract, offering protection against infections, modulating tumor immunity, and influencing chronic inflammatory diseases. While their ability to provide long-lasting, localized immunity is promising, their dysregulation can contribute to chronic inflammation and tissue damage, especially in conditions like interstitial cystitis or chronic prostatitis. The therapeutic potential of T_RM_ cells is immense, particularly in developing vaccines, enhancing cancer immunotherapy, and targeting chronic inflammation. Beyond urinary tract cancers, T_RM_ cells have also been implicated in other malignancies, including lung cancer, breast cancer, and colorectal cancer. In non-small cell lung cancer, T_RM_ cells enriched in tumor tissues have been associated with improved responses to ICIs, particularly PD-1/PD-L1 blockade. Similarly, in breast cancer, T_RM_ cells contribute to local immune surveillance, though their exact role varies across molecular subtypes. In colorectal cancer, T_RM_ cells have been linked to enhanced tumor control and better prognosis, especially in microsatellite instability-high tumors. Future research should explore how T_RM_ cells contribute to tumor immunity across different cancer types and how their functional states may vary depending on the tumor microenvironment. Understanding the mechanisms regulating T_RM_ cells exhaustion and activation in diverse cancers could provide insights into optimizing T_RM_-targeted immunotherapies. Expanding research beyond urinary tract cancers will be crucial for fully harnessing the therapeutic potential of T_RM_ cells in oncology. Future research should focus on understanding the complex interactions of T_RM_ cells in different disease contexts and exploring strategies to harness their protective functions while mitigating their pathological effects. By doing so, we can develop more effective and personalized treatments for urinary tract diseases.
